# Contributions to the systematics of New World macro-moths IV

**DOI:** 10.3897/zookeys.264.4687

**Published:** 2013-02-06

**Authors:** B. Christian Schmidt, J. Donald Lafontaine

**Affiliations:** 1 Canadian Food Inspection Agency, Canadian National Collection of Insects, Arachnids and Nematodes, K.W. Neatby Bldg., 960 Carling Ave., Ottawa, ON, Canada K1A 0C6; 2 Canadian National Collection of Insects, Arachnids, and Nematodes, Biodiversity Program, Agriculture and Agri-Food Canada, K.W. Neatby Bldg., C.E.F., Ottawa, Ontario, Canada K1A 0C6

This special issue of ZooKeys, “Contributions to the systematics of New World macro-moths IV” marks the fourth volume in this series, initiated in May 2009 (ZooKeys # 9), with the second volume published in March 2010 (ZooKeys #39), and the third volume published in November 2011 (Schmidt and Lafontaine 2009, 2010, 2011). Twenty-two authors contributed 12 manuscripts for this volume, covering taxa in the Crambidae, Erebidae, Euteliidae, Geometridae, Noctuidae, and Notodontidae. New taxa are described from Argentina, Bahamas, Bolivia, Brazil, Canada, Cayman Islands, Colombia, Costa Rica, Cuba, Dominica, Dominican Republic, Ecuador, French Guiana, Guadeloupe, Guatemala, Guyana, Haiti, Honduras, Jamaica, Paraguay, Peru, Puerto Rico, British Virgin Islands, U.S. Virgin Islands, St. Lucia, Suriname, Trinidad, United States, and Venezuela. Taxonomic changes include the description of 27 new species and four new subspecies, eight new or revised synonyms, two revised statuses, and one new generic combination.

Since its inception in 2009, the “Contributions” series collectively includes 49 taxonomic publications by 38 authors. Geographic coverage has previously focused primarily on the North American fauna (Canada, United States and Mexico).

 This issue marks a considerable increase in contributions concerning Neotropical taxa, and we look forward to continued emphasis of both realms in future volumes. Authors interesting in contribution to future editions of “Contributions …” are encouraged to contact us.
